# High Prevalence of Vitamin D Deficiency among Pregnant Saudi Women

**DOI:** 10.3390/nu8020077

**Published:** 2016-02-04

**Authors:** Nora A. Al-Faris

**Affiliations:** Nutrition and Food Science Department, College of Home Economics, Princess Nourah Bint Abdulrahman University, P.O. Box 27938, Riyadh 11427, Saudi Arabia; naalfaris@pnu.edu.sa; Tel.: +966-1182-37437

**Keywords:** vitamin D, deficiency, pregnancy, women, Saudi Arabia

## Abstract

Vitamin D deficiency has emerged as a public health problem worldwide due to its important role in health and disease. The present work is intended to examine prevalence of vitamin D deficiency among pregnant Saudi women and related risk factors. A cross-sectional study was carried out at King Fahad Medical City in Riyadh, Saudi Arabia. Serum 25-hydroxy vitamin D (25(OH)D) was measured by enzyme-linked immunosorbent assay in 160 pregnant women during the first trimester of pregnancy. Socio-demographic, lifestyle and maternal characteristics were collected and vitamin D intake was assessed using a 24-h dietary recall. Weight and height were measured using standardized methods. Vitamin D deficiency (25(OH)D < 50 nmol/L) and insufficiency (25(OH)D = 50–74 nmol/L) were reported in 50% and 43.8% of the study sample, respectively. Median serum 25(OH)D concentration was 49.9 nmol/L. Adequate vitamin D intake (≥600 IU/day) was reported among only 8.1% of pregnant women. Age group, educational level, sun exposure frequency and daytime and daily practice of exercise were significantly associated with vitamin D status. Overall, vitamin D deficiency was common among pregnant Saudi women in Riyadh. Steps should be taken to address the current situation, including increased sunlight exposure, consumption of fatty fish, and vitamin D supplements.

## 1. Introduction

Vitamin D is an essential fat-soluble vitamin that is required for regulation of calcium metabolism and to maintain good health [1]. It is obtained through either dietary sources or synthesis in the human skin by exposure to ultraviolet B (UVB) radiation [2]. There are two main forms of vitamin D: vitamin D_2_ (ergocalciferol) and vitamin D_3_ (cholecalciferol). Vitamin D_2_ is a plant sterol found in foods such as cod-liver oil, fatty fish, and egg yolk. Vitamin D_3_ is manufactured in human skin through photochemical conversion of 7-dehydrocholesterol via pre-vitamin D_3_ to vitamin D_3_. In the human body, vitamin D is converted to 25-hydroxy vitamin D (25(OH)D), the major storage and circulating form of vitamin D, and then to 1,25-dihydroxy vitamin D, the active form of vitamin D, by enzymes in the liver and kidney [2].

Vitamin D deficiency has been recognized as an international public health problem due to its important role in health and disease, mainly for the skeletal system where vitamin D deficiency causes rickets, osteomalacia, and osteoporosis [3]. Even in countries with plentiful sunshine, epidemic prevalence of vitamin D deficiency has been reported in the general population and especially in women and children [4,5]. Pregnant women are more susceptible to vitamin D deficiency than any other human groups [6]. The current evidence emphasizes that vitamin D deficiency during pregnancy is accompanied with several adverse maternal and neonatal outcomes, including elevated risks of gestational diabetes, pre-eclampsia, caesarean section, postpartum depression, preterm birth, and low birth weight [7,8]. Fortunately, vitamin D status is a modifiable factor. Therefore, it is important to determine factors associated with vitamin D status during pregnancy [9].

This study was conducted to investigate the prevalence of vitamin D deficiency among a sample of pregnant Saudi women. Moreover, the study aimed to determine factors associated with vitamin D deficiency during pregnancy, including socio-demographic, lifestyle, and maternal characteristics, as well as vitamin D intake. It was based on two hypotheses. First, vitamin D deficiency is common among pregnant Saudi women. Second, their vitamin D intake is low.

## 2. Materials and Methods

### 2.1. Study Design and Population

The present work is a cross-sectional study conducted during the spring of 2010 (March to May). The eligibility criteria and target population of this study is comprised of pregnant Saudi women in the first trimester of pregnancy, aged 20 to 49 years, and living in Riyadh; the capital city of Saudi Arabia (latitude 24.7°N). The study sample was drawn by systematic random sampling method from all eligible patients who came for a follow-up examination at the gynecology clinic at King Fahad Medical City in Riyadh, Saudi Arabia during the study period. Patients’ data were obtained from the hospital medical records registry. Totally, 160 pregnant women were successfully recruited to participate in this study. The response rate was 80%. Electronic medical records of all participants were checked to confirm that they had not been diagnosed with any medical disorder that interferes with vitamin D status. Prior to taking part in this study, all participants signed a consent form in accordance with the declaration of Helsinki. The study protocol was approved by external research review committee at King Fahad Medical City in Riyadh, Saudi Arabia.

### 2.2. Data Collection

Face-to-face interviews were used to collect data by trained interviewers. Collected data include socio-demographic and lifestyle characteristics such as age, address, and sun exposure and maternal characteristics such as gravidity, parity and inter-pregnancy intervals. The sun exposure and daily practice of exercise of subjects were self-reported depend on their occupational and recreational outdoor activities. The level of sun exposure was defined as rare, sometimes or frequent when they exposed at least 15% of their bodies (e.g., face, arms and hands) for at least 15 min to the sun once or less, twice, or at least three times a week, respectively. For exercise practice, engaging in a moderate level of physical activity, such as walking, for at least 60 min, once or less, twice, or at least three times per week were defined as rare, sometimes or frequent daily practice of exercise, respectively. Weight and height were measured using standardized methods by the gynecologist during the consultation. Therefore, maternal body mass index (BMI) was calculated as the ratio of weight (kg) to height (m^2^). Participants’ BMI was then categorized into: underweight (<18.5), normal weight (18.5–24.9), overweight (25–29.9) and obese (≥30). In addition, vitamin D intake was assessed using a 24-h dietary recall for the previous day. Specific details related to the types and amounts of consumed foods, preparation methods, and serving size for each item were gathered. A nutrient analysis software program (Food Processor for Windows, version 7.71, ESHA Research, Salem, OR, USA) was used to estimate the daily intake of vitamin D for each participant. Vitamin D intake was considered adequate when daily intake reached 600 IU (0.015 mg) or more according to dietary reference intakes (DRIs) for pregnant women [10].

### 2.3. Blood Samples and Laboratory Analysis

Fasting blood samples were collected from the antecubital vein of all enrolled pregnant women at the first prenatal consultation visit by registered nurse. Samples (*n* = 160) were taken directly to the clinical laboratory at King Fahad Medical City, Riyadh, for serum 25(OH)D concentrations assay. The test was performed using the Roche Cobas e601 immunoassay analyzer using the Roche Elecsys vitamin D_3_ assay (Roche Diagnostics, Mannheim, Germany). Vitamin D status was categorized as sufficient (25(OH)D ≥ 75 nmol/L), insufficient (25(OH)D = 50–74 nmol/L), and deficient (25(OH)D < 50 nmol/L). Furthermore, serum 25(OH)D concentration less than 25 nmol/L was considered severe vitamin D deficiency [11].

### 2.4. Statistical Analysis

The Statistical Package for Social Sciences (SPSS Inc., Chicago, IL, USA) version 20 was used for data analysis. Categorical variables were expressed as frequencies and percentages, and analyzed using a chi-square test. Continuous variables were expressed as medians and interquartile ranges (IQR) as they were not normally distributed. Univariate and multivariate logistic regression analysis were conducted to identify factors associated with vitamin D deficiency, after adjusting for age, BMI, and vitamin D intake. All reported *p* values were made on the basis of two-tailed tests. Differences were considered statistically significant at *p* < 0.05.

## 3. Results

In total, 160 pregnant Saudi women participated in the current study. Most of them (78.8%) were aged 20–34 years, while the rest were aged 35–49 years (Table 1). 

**Table 1:** Socio-demographic and lifestyle characteristics and the prevalence of vitamin D deficiency among participants.

Variables	Participants *N* (%)	Vitamin D Deficiency (%)	*p*-Value *
Age Groups			
20–34 years	126 (78.8%)	53.2%	0.029
35–49 years	34 (21.3%)	38.2%	
Address in Riyadh City			
East of city	43 (26.9%)	62.8%	0.11
West of city	29 (18.1%)	55.2%	
North of city	38 (23.8%)	47.4%	
South of city	50 (31.3%)	38.0%	
Type of Housing			
House	76 (47.5%)	50.0%	0.699
Apartment	84 (52.5%)	50.0%	
Family Income			
Less than 1000 USD	51 (31.9%)	43.1%	0.826
1000–2000 USD	70 (43.8%)	52.9%	
More than 2000 USD	39 (24.4%)	53.8%	
Education Level			
High school education or less	93 (58.1%)	44.1%	0.049
College education or more	67 (41.9%)	58.2%	
Employment Status			
No	133 (83.1%)	48.1%	0.489
Yes	27 (16.9%)	59.3%	
Sun exposure			
Rarely	70 (43.8%)	48.6%	0.014
Sometimes	57 (35.6%)	54.4%	
Frequently	33 (20.6%)	45.5%	
Daytime of sun exposure **			
Morning	37 (25.9%)	67.6%	0.001
Midday	52 (36.4%)	55.8%	
Evening	54 (37.8%)	27.8%	
Daily sun exposure duration **			
Less than 15 min	117 (81.8%)	46.2%	0.512
15 min or more	26 (18.2%)	57.7%	
Daily practice of exercise			
Rarely	51 (31.9%)	60.8%	0.016
Sometimes	42 (26.3%)	50.0%	
Frequently	67 (41.9%)	41.8%	

* Categorical variables were expressed as numbers and percentages, and analyzed using a chi-square test. Differences were considered statistically significant at *p* < 0.05. ** Data for 17 participants were missing.

Participants were living in the different regions of Riyadh city. In fact, 26.9%, 18.1%, 23.8% and 31.3% of them came, respectively, from east, west, north and south of the city. Moreover, they live with their families in either apartments (52.5%) or houses (47.5%). Monthly family income was low (less than 1000 USD) for 31.9% of the study sample, medium (1000–2000 USD) for another 43.8% of the participants and high (more than 2000 USD) for the rest of them. Furthermore, 67 pregnant women (41.9%) have at least a college degree and only 27 participants (16.9%) have a job. While 43.8% of the participants were rarely exposed to the sun, only 20.6% of them had frequent sun exposure (data for 17 participants were missing). The usual time of sun exposure was midday (36.4%) and evening (37.8%) and the duration of sun exposure for 81.8% of the participants did not exceed 15 min daily. Additionally, frequent daily practice of exercise was reported in 67 women (41.9%).

As shown in Table 2, 35 women (21.9%) were pregnant for the first time (primagravida) and the rest of the participants were multigravida. In addition, 21.9% of women have not given birth previously (nulliparous), while 34.4% and 43.8% of them were primiparous and multiparous, respectively. Inter-pregnancy intervals were two years or less for about half of pregnant women (50.4%) when primagravida women were excluded (*n* = 35). During previous pregnancy, 99 women (79.2%) had normal birth while 26 women (20.8%) underwent to caesarean birth (35 women were nulliparous women and therefore were excluded). Based on maternal BMI, most of the participants were overweight (51.6%) or obese (26.1%) (BMI data for seven participants were missing). The vast majority of pregnant women (91.9%) had inadequate vitamin D intake, as their daily intake levels did not reach the recommended levels (600 IU/day) (Table 2). In contrast, only thirteen women (8.1%) achieved vitamin D adequacy. Vitamin D was not taken by any participants as a single nutrient supplement. Instead, 55% of women used multi-vitamin supplements that contained varying amounts of vitamin D (on average 400 IU/tablet). The other supplementations reported by pregnant women were folate (83.1%), iron (74.4%), calcium (59.4%), zinc (6.9%), and multi-minerals (25.6%) supplements.

**Table 2:** Maternal characteristics, vitamin D and dietary supplements intake and the prevalence of vitamin D deficiency among participants.

Variables	Participants *N* (%)	Vitamin D Deficiency (%)	*p*-Value *
Gravidity (Number of pregnancies)			
Primagravida	35 (21.9%)	57.1%	0.195
Multigravida	125 (78.1%)	48.0%	
Parity (Number of deliveries)			
Nulliparous	35 (21.9%)	57.1%	0.317
Primiparous	55 (34.4%)	45.5%	
Multiparous	70 (43.8%)	50.0%	
Inter-pregnancy intervals **			
2 years or less	63 (50.4%)	46.0%	0.788
More than 2 years	62 (49.6%)	50.0%	
Previous method of birth ***			
Normal birth	99 (79.2%)	50.5%	0.235
Caesarean birth	26 (20.8%)	38.5%	
Maternal BMI status ****			
18.5–25	34 (22.2%)	41.2%	0.551
25–29.9	79 (51.6%)	51.9%	
≥ 30	40 (26.1%)	50.0%	
Vitamin D intake			
Inadequate intake	147 (91.9%)	51.0%	0.319
Adequate intake	13 (8.1%)	38.5%	
Multi-vitamin supplement			
No	72 (45.0%)	54.2%	0.224
Yes	88 (55.0%)	46.6%	

* Categorical variables were expressed as numbers and percentages, and analyzed using a chi-square test. Differences were considered statistically significant at *p* < 0.05. ** Primagravida women were excluded (*n* = 35). *** Nulliparous women were excluded (*n* = 35). **** BMI data for seven participants were missing.

**Figure 1:**
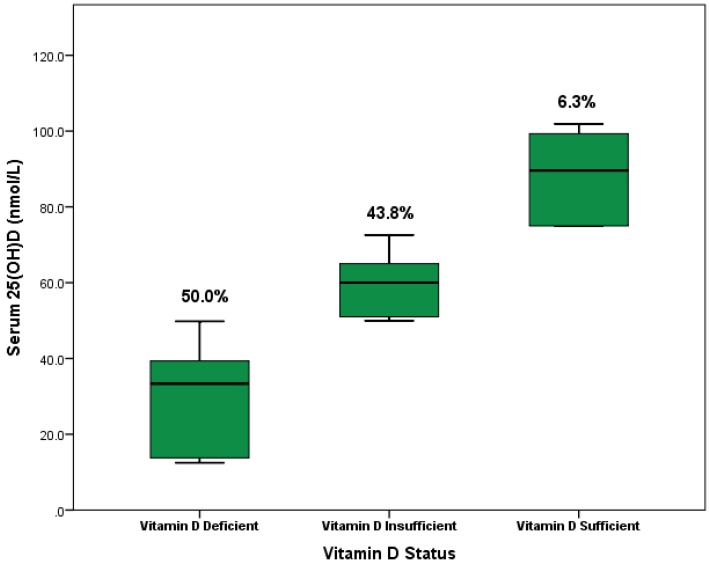
Boxplot chart illustrating serum 25(OH)D levels according to vitamin D status among 160 pregnant Saudi women; 80 participants were vitamin D deficient (50.0%), 70 participants were vitamin D insufficient (43.8%) and 10 participants were vitamin D sufficient (6.3%).

The overall prevalence of vitamin D deficiency (25(OH)D < 50 nmol/L) in pregnant women was 50% (Figure 1). Among them, 28 women (18.1%) had severe vitamin D deficiency (25(OH)D < 25 nmol/L). Furthermore, vitamin D insufficiency (25(OH)D = 50–74 nmol/L) was reported in 43.8% of the study sample. However, only 6.3% of the participants were diagnosed with vitamin D sufficiency (25(OH)D ≥ 75 nmol/L). The median of serum 25(OH)D concentrations of all participants was 49.9 nmol/L (IQR = 28.0 nmol/L) and the highest reading recorded among participants was 101.9 nmol/L (Range = 89.4). The median of serum 25(OH)D concentrations among women with vitamin D deficiency, insufficiency and sufficiency were 33.4 nmol/L, 60.0 nmol/L and 89.6 nmol/L, respectively (Figure 1).

Younger pregnant women (aged 20–34 years) had a significantly (*p*-value = 0.029) higher prevalence (53.2%) of vitamin D deficiency compared with older women (35–49 years, 38.2%) (Table 1). Surprisingly, educated women with at least a college degree had a significantly (*p*-value = 0.049) higher prevalence (58.2%) of vitamin D deficiency than those with lower educational level (44.1%). Moreover, subjects with frequent sun exposure had a significantly (*p*-value = 0.014) lower prevalence of vitamin D deficiency compared with subjects with less frequent sun exposure. Similarly, subjects usually exposed to sun in the evening had a significantly (*p*-value = 0.001) lower prevalence (27.8%) of vitamin D deficiency than subjects exposed to sun in the morning (67.6%) and midday (55.8%). Interestingly, the daily practice of exercise was significantly associated with vitamin D status (*p*-value = 0.016). Vitamin D deficiency level decreased as the daily practice of exercise increased. However, other socio-demographic and lifestyle variables were not associated with vitamin D deficiency. Although the association between maternal characteristics and vitamin D deficiency was not significant, primagravida and nulliparous women had a higher prevalence (57.1%) of vitamin D deficiency than others (Table 2). In the same manner, overweight and obese women had a higher prevalence (51.9% and 50.0%, respectively) of vitamin D deficiency compared to those with normal body weight (41.2%). Pregnant women with inadequate vitamin D intake levels had a higher prevalence (51%) of vitamin D deficiency compared with those with adequate vitamin D intake levels (38.5%), but the difference was not significant (*p*-value = 0.319) (Table 2).

In multivariate analysis, as presented in Table 3, the only variables that were significantly associated with low vitamin D status were education level and time of day of sun exposure. Compared to educated pregnant women, less educated women had higher odds of vitamin D deficiency status (adjusted odds ratio (OR) = 2.00, *p* = 0.042). Sun exposure at the end of the day was associated with lower risk for vitamin D deficiency compared to sun exposure at the beginning of the day (adjusted OR = 4.93, *p* = 0.001) and midday (adjusted OR = 3.37).

**Table 3:** Risk of vitamin D deficiency among participants for selected variables.

Variables	Participants *N* (%)	Unadjusted Odds Ratio (95% CI)	*p*-Value *	Adjusted Odds Ratio ** (95% CI)	*p*-Value *
Age Groups					
20–4 years	126 (78.8%)	1	0.125	1	0.155
35–49 years	34 (21.3%)	0.55		0.56	
Education Level					
High school education or less	93 (58.1%)	1	0.079	1	0.042
College education or more	67 (41.9%)	1.77		2.00	
Sun exposure					
Rarely	70 (43.8%)	1.13	0.682	1.16	0.541
Sometimes	57 (35.6%)	1.43		1.60	
Frequently	33 (20.6%)	1		1	
Daytime of sun exposure					
Beginning of the day	37 (25.9%)	5.42	0.001	4.93	0.001
Midday	52 (36.4%)	3.28		3.37	
End of the day	54 (37.8%)	1		1	
Daily practice of exercise					
Rarely	51 (31.9%)	2.16	0.127	2.50	0.066
Sometimes	42 (26.3%)	1.39		1.78	
Frequently	67 (41.9%)	1		1	
Gravidity (Number of pregnancies)					
Primagravida	35 (21.9%)	1	0.340	1	0.511
Multigravida	125 (78.1%)	0.69		0.77	
Parity (Number of deliveries)					
Nulliparous	35 (21.9%)	1	0.559	1	0.639
Primiparous	55 (34.4%)	0.63		0.67	
Multiparous	70 (43.8%)	0.75		0.87	
Maternal BMI status					
18.5–25	34 (22.2%)	1	0.575	1	0.417
25–29.9	79 (51.6%)	1.54		1.74	
≥30	40 (26.1%)	1.43		1.63	
Vitamin D intake					
Inadequate intake	147 (91.9%)	1.67	0.389	1.48	0.528
Adequate intake	13 (8.1%)	1		1	
Multi-vitamin supplement					
No	72 (45.0%)	1	0.341	1	0.225
Yes	88 (55.0%)	0.74		0.66	

* Differences were considered statistically significant at *p* < 0.05. ** Groups analyzed using multivariate logistic regression analysis after adjusting for age, BMI, and vitamin D intake.

## 4. Discussion

To our knowledge, the current work is the first attempt to explore the prevalence of vitamin D deficiency among pregnant Saudi women in Riyadh city during the last three decades. This study found a high prevalence of vitamin D deficiency and insufficiency among pregnant Saudi women. Over 90% of pregnant women in this study were either vitamin D deficient or insufficient. Vitamin D deficiency or insufficiency is not expected to be prevalent in Saudi Arabia as a country with abundant sunlight throughout the year [12]. However, several studies have reported a high prevalence of vitamin D deficiency among Saudi population of different age groups with higher prevalence among females [13,14,15,16]. The early reports of a potential vitamin D deficiency among Saudi women appeared about 30 years ago. Two epidemiologic studies published in the early 1980s noticed low serum 25(OH)D concentrations among pregnant and non-pregnant Saudi women [17,18]. Kanan and colleagues [19] investigated vitamin D status among 1556 Saudi women aged 19 years or more during the summer and winter seasons. The high prevalence of vitamin D deficiency was reported among both premenopausal and postmenopausal women (80% and 68%, respectively) during both seasons. In another study which was conducted on 465 Saudi women aged 19–40 years and are living in Riyadh, Al-Mogbel [20] reported that all participants were vitamin D deficient (serum 25(OH)D < 50 nmol/L) and about 79% of them had severe vitamin D deficiency (serum 25(OH) D < 25 nmol/L). A survey conducted among 1172 healthy Saudi women in Jeddah, the western region of Saudi Arabia, showed that 80% of them exhibited vitamin D deficiency (serum 25(OH)D < 50 nmol /L) [21]. A limited number of studies have been published on the assessment of vitamin D levels among pregnant Saudi women. Azhar [22] determined serum 25(OH)D in 118 healthy pregnant and non-pregnant women from the western region of Saudi Arabia. Results showed that 97.5% of women (95.8% of pregnant women and 98.6% of non-pregnant women) had 25(OH)D concentrations equal to or less than 50 nmol/L, which indicate that most of these women were vitamin D deficient.

Vitamin D status is usually affected by several determinants, especially those that have an impact on the dermal synthesis rate of vitamin D. For example, latitude, season of the year, time of day and air pollution affect the amount of UVB radiation reaching the earth's surface and therefore skin vitamin D production [2,3]. In fact, factors that are associated with vitamin D status in Saudi Arabia often relate to Saudi customs. Saudi Arabia has ample sunshine throughout the year, and temperatures during the summer months often rise above 50 °C. Therefore, Saudi people usually limit their outdoors activities during daytime. Moreover, most Saudi women wear dark veils that cover their bodies completely, which block sunlight, due to cultural and religious reasons [20,22,23]. This applies to the current study participants who wear dark veils that cover their bodies completely.

Dietary sources of vitamin D include foods that contain vitamin D, foods that are fortified with vitamin D, and vitamin D supplements. Most foods do not contain significant amounts of vitamin D to achieve vitamin adequacy. Fatty fish such as salmon, tuna and sardines provide 200–350 IU of vitamin D per 100 grams. Eggs contain approximately 20 IU of vitamin D per egg. Cereal products usually provide 40–50 IU of vitamin D per cup [22,24]. In Saudi Arabia, lifestyles and dietary patterns have changed dramatically toward sedentary lifestyles focusing mainly on indoor activities and energy-dense foods such as fast food at the expense of nutrients-dense foods such as fruits, vegetables, whole grains and fat free dairy products. All of this leads to a decrease in vitamin D intake levels [23,25,26]. Fish consumption is not popular in Saudi community, in contrast to red meat and poultry, especially in landlocked cities such as Riyadh [20]. Typical vitamin D fortified foods include milk, buttermilk, and yogurt. Fortunately, these fortified dairy products are available in the Saudi market and provide about 40–400 IU of vitamin D per liter [24]. However, present fortification level of dairy products is too low to prevent vitamin D deficiency [19]. Sadat-Ali *et al.* found that in Saudi Arabia, most commonly consumed foods which are supposed to be vitamin D fortified were either not fortified or contain an amount less than recommended by guidelines set for the US marketplace [24]. Vitamin D supplements consumption is uncommon among Saudi women [15]. In most public hospitals in Saudi Arabia, vitamins are prescribed for people who have health conditions. Pregnant Saudi women have poor vitamin D supplementation practices and usually consume vitamins that are necessary for maternal and neonate health such as folic acid and iron [22,27]. The Saudi Osteoporosis Society (SOS) recommended pregnant women to consume daily 600 IU of vitamin D supplements [28].

Our results revealed that younger women had a higher prevalence of vitamin D deficiency than older women. Several studies reported the same result [14,16,20]. Kanan *et al.* [20] reported that women younger than 30 years old were more vitamin D deficient compared to those older than 30 years old. This could be due to more common vitamin supplements taken by older women and unhealthy dietary pattern among younger women characterized by high fast food consumption [26]. Furthermore, more educated pregnant women in this study had a significantly higher prevalence of vitamin D deficiency compared with less educational women. Another study reported the same finding. Their explanation for this finding was based on the fact that most highly educated women are employed indoors with less sun exposure and less healthy diets comprised mainly of fast food, compared to housewives who have more free time for sun exposure and better dietary pattern [20]. As expected, we find that women with frequent sun exposure had a significantly lower prevalence of vitamin D deficiency compared with subjects with less sun exposure frequency. This supports previous findings [20,29]. In one study conducted in Riyadh, Saudi Arabia, 47 volunteers were given verbal advice to expose themselves to sunlight for 5–30 min twice weekly and were encouraged to increase their dietary vitamin D intake. A significant decrease in the prevalence of vitamin D deficiency from 44% to 27% was observed at one-year follow-up [29]. Unexpectedly, our results discovered that women usually exposed to the sun in the evening had a significantly lower prevalence of vitamin D deficiency than those exposed to sun in the morning and midday. Sensible sun exposure, which depends on several determinants, such as latitude, season, skin pigmentation and daytime, is essential to providing an adequate amount of vitamin D [3]. Usually, midday, between the hours of 10:00 a.m. and 3:00 p.m., is often the ideal time of day to achieve maximum vitamin D production [3]. However, Saudi Arabia is a sunny place and temperatures can be dangerously hot, rising above 50 °C in non-winter months [12]. Consequently, it is common for the Saudi people to avoid spending time outdoors in the middle of the day, which could make the evening better choice for them to have sensible sun exposure. The vitamin D deficiency level was decreased significantly as the daily practice of exercise based on outdoor activities increased among participants in the current study. In a recent study, 65 Saudi women were recruited for one year into three groups. The first group received only advice for healthy food, while the second group received the same advice in addition to vitamin D supplements. The third group received exercise in a sport center in combination with advice for healthy food and vitamin D supplements. Results revealed that the first group had no significant change in the level of serum vitamin D. Vitamin D level in the second group increased up to 70% of the base readings. Interestingly, vitamin D level of third group increased up to 300% of the initial readings [30]. In the present study, overweight and obese women are more likely to be vitamin D deficient. This is consistent with similar studies reported that a strong relationship observed between obesity and vitamin D deficiency [14,15,31]. The suggested mechanism behind this association is that elevated concentrations of 1,25-dihydroxy vitamin D stimulate lipogenesis and inhibit lipolysis in cultured human adipocytes, leading to accumulation of fat [32]. Moreover, 1,25-dihydroxy vitamin D inhibits the expression of adipocyte uncoupling protein 2 (UCP2), which would cause a reduction in the adipocyte’s metabolic efficiency [33].

Generally, Saudi women were limited in their knowledge about vitamin D and vitamin D deficiency [34]. They had limited sun exposure due to elevated temperatures, and covering their bodies completely for cultural reasons that makes sun exposure difficult [34]. Governmental actions should be taken to address the current situation. Increasing awareness of vitamin D importance and guidelines to obtain adequate vitamin D level, creating areas where women, particularly those of lower socio-economic status, can reach sun exposure as well as more food fortification could be helpful in addressing this serious problem [34]. Our study had a number of limitations. First, the sample size was small. Second, variations in vitamin D status across different pregnancy trimesters, and different year seasons were not detected as we conduct our study on first trimester pregnant women during the spring season. Finally, the assay of vitamin D was performed using immunoassay technique, which may overestimate results [35]. However, this is the first study, to our knowledge, that investigates the prevalence of vitamin D deficiency among pregnant Saudi women in Riyadh city during last three decades.

## 5. Conclusions

In conclusion, vitamin D deficiency was common among pregnant Saudi women who reside in Riyadh. We believe that more attention should be given to vitamin D status among pregnant women because it is an important vitamin for regulation of calcium metabolism and maintain good health, which is unfortunately lacking in most of pregnant Saudi women. Several steps could be taken to address the current situation. Sunlight exposure needs to be increased for these women by encouraging them to involve in more outdoor activity during the daytime. They should expose their skin (e.g., face, arms and hands) to the sun to produce sufficient levels of vitamin D for optimal overall health. Vitamin D fortification of dairy products that are available in the local market and fatty fish consumption should be increased since these foods are good sources of vitamin D. In addition, dietary supplements of vitamin D should be considered for people at risk of vitamin D deficiency such as pregnant women in order to avoid deficiency health-related outcomes. Finally, effective education programs at the national level targeted pregnant Saudi women are needed to elevate the public awareness of this serious problem.
